# A New Modified CKD-EPI Equation for Chinese Patients with Type 2 Diabetes

**DOI:** 10.1371/journal.pone.0109743

**Published:** 2014-10-14

**Authors:** Xun Liu, Xiaoliang Gan, Jinxia Chen, Linsheng Lv, Ming Li, Tanqi Lou

**Affiliations:** 1 Division of Nephrology, Department of Internal Medicine, The Third Affiliated Hospital of Sun Yat-sen University, Guangzhou, China; 2 Department of Anesthesiology, Zhongshan Ophthalmic Center, Sun Yat-sen University, Guangzhou, China; 3 Operating Room, The Third Affiliated Hospital of Sun Yat-sen University, Guangzhou, China; University of Utah School of Medicine, United States of America

## Abstract

**Objective:**

To improve the performance of glomerular filtration rate (GFR) estimating equation in Chinese type 2 diabetic patients by modification of the CKD-EPI equation.

**Design and patients:**

A total of 1196 subjects were enrolled. Measured GFR was calibrated to the dual plasma sample ^99m^Tc-DTPA-GFR. GFRs estimated by the re-expressed 4-variable MDRD equation, the CKD-EPI equation and the Asian modified CKD-EPI equation were compared in 351 diabetic/non-diabetic pairs. And a new modified CKD-EPI equation was reconstructed in a total of 589 type 2 diabetic patients.

**Results:**

In terms of both precision and accuracy, GFR estimating equations all achieved better results in the non-diabetic cohort comparing with those in the type 2 diabetic cohort (30% accuracy, P≤0.01 for all comparisons). In the validation data set, the new modified equation showed less bias (median difference, 2.3 ml/min/1.73 m^2^ for the new modified equation vs. ranged from −3.8 to −7.9 ml/min/1.73 m^2^ for the other 3 equations [P<0.001 for all comparisons]), as was precision (IQR of the difference, 24.5 ml/min/1.73 m^2^ vs. ranged from 27.3 to 30.7 ml/min/1.73 m^2^), leading to a greater accuracy (30% accuracy, 71.4% vs. 55.2% for the re-expressed 4 variable MDRD equation and 61.0% for the Asian modified CKD-EPI equation [P = 0.001 and P = 0.02]).

**Conclusion:**

A new modified CKD-EPI equation for type 2 diabetic patients was developed and validated. The new modified equation improves the performance of GFR estimation.

## Introduction

Diabetic nephropathy is the leading cause of end stage renal disease and is associated with significantly high risk of cardiovascular events [1]. Glomerular filtration rate (GFR) is the best index of kidney function.[2]. American Diabetes Association standards highlight GFR screening for nephropathy in diabetic patients [3]. The Modification of Diet in Renal Disease (MDRD) equation and the Chronic Kidney Disease Epidemiology Collaboration (CKD-EPI) equation are most frequently used and favored in North America, Europe and Australia [4]. However, when either equation was applied to type 2 diabetic patients, both have imperfections [5]–[8], because of the intrinsic factors such as serum glucose status [9] and body mass index [10] in diabetic subjects that could affect the accuracy of GFR estimates. Recently, a four-level race variable (Black, Asian, Native American and Hispanic, and White and other) CKD-EPI equation [11] was developed. In order to know whether the most frequently used equations really performed worse in diabetic subjects, a well-designed paired cohort was set up in this study to exclude other impact factors. And if this hypothesis was confirmed, a new equation was reconstructed later by modification of the original GFR estimating equation in a cohort of type 2 diabetic patients.

## Subjects, Materials and Methods

### Participant selection

This study enrolled participants consequently from Jan 2010 to Dec 2012 in the Third Affiliated Hospital of Sun Yat-sen University, China. Participants were excluded if they had any of the following: 1) younger than 18 years, or 2) type 1 diabetes, or 3) type 2 diabetes with known non-diabetic renal disease. The other exclusion criteria were described elsewhere [12]. GFR category was classified according to the National Kidney Foundation Disease Outcomes Quality Initiative clinical practice guidelines [13]. A total of 1196 subjects were enrolled, including 589 type 2 diabetic patients and 607 non-diabetic participants. The study protocol was approved by the institutional review board at the Third Affiliated Hospital of Sun Yat-sen University. Written informed consent was obtained from each participant.

### Laboratory methods

GFR was measured by the 99mTc-diethylene triamine pentaacetic acid (^99 m^Tc-DTPA) renal dynamic imaging method [14]–[15], as described previously [16]. The minimum sample size was determined to be as 36 based in the findings in a previous study [17]. The calibration equation form DTPA renal dynamic imaging GFR to dual plasma sample DTPA-GFR in this study was as the following: dual plasma sample DTPA-GFR (ml/min/1.73 m^2^) =  0.167+1.057* DTPA renal dynamic imaging-GFR (ml/min/1.73 m^2^) (R^2^  =  0.767, P<0.001). Serum creatinine (SC) level was measured by the enzymatic method on a Hitachi 7180 autoanalyzer (Hitachi, Tokyo, Japan; reagents from Roche Diagnostics, Mannheim, Germany), and recalibrated to isotope dilution mass spectrometry.

### Statistical analysis

We used a stratified random sampling method based on age, body mass index (BMI) and GFR categories to obtain paired samples of participants represented either the type 2 diabetes or the non-diabetic cohorts. The bias between mGFR and estimated GFR (eGFR) was defined as mGFR minus eGFR. Precision was measured as the interquartile range (IQR) for difference. Accuracy was determined as the percentage of eGFR not deviating more the 30% from the mGFR. Confidence intervals for all metrics were calculated by means of bootstrap methods [18]. A Wilcoxon Mann-Whitney test was used for bias. In comparison between two data sets, independent samples t test was used for quantitative variables, and two independent samples test for accuracy. In comparison within a data set, McNemar test was used for accuracy. GFR was estimated by using the following equations: re-expressed 4-variable MDRD equation (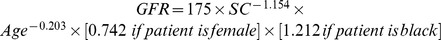) [19], CKD-EPI equation (Table 1) [20] and Asian modified CKD-EPI equation (Table 1) [11]. All analyses were performed using SPSS software (version 11.0 SPSS, Chicago IL, USA) and Matlab software (version 2011b The Mathworks, Boston MA, USA).

**Table 1:** CKD-EPI equation, asian modified CKD-EPI equation and the new equation.

Basis of equation and sex	Serum creatinine	Equation for estimating GFR
CKD-EPI equation		
Female	≤ 0.7 mg/dl	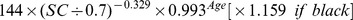
Female	> 0.7 mg/dl	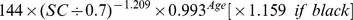
Male	≤ 0.9 mg/dl	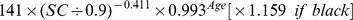
Male	> 0.9 mg/dl	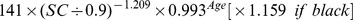
Asian modified CKD-EPI equation		
Female	≤ 0.7 mg/dl	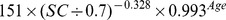
Female	> 0.7 mg/dl	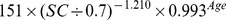
Male	≤ 0.9 mg/dl	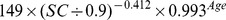
Male	> 0.9 mg/dl	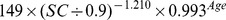
New modified CKD-EPI equation		
Female	≤ 0.7 mg/dl	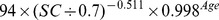
Female	> 0.7 mg/dl	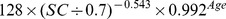
Male	≤ 0.9 mg/dl	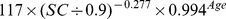
Male	> 0.9 mg/dl	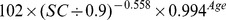

## Results

### Performance of the equations between diabetic and non-diabetic cohorts

#### Study population in this part of study

Three hundred and fifty-one pair of participants were selected from the total population of this study. In the type 2 diabetic cohort, the mean (±SD) mGFR was 62.8±28.1 ml/min/1.73 m^2^. The mean mGFR was similar in the non-diabetic cohort (60.7±27.9 ml/min/1.73 m^2^), as were the mean age, BMI, body-surface area, SC and gender (Table 2).

**Table 2:** Participant characteristic.*

Subjects (n)	Participant group		P value
	Non-diabetics (n = 351)	Diabetics (n = 351)	
Age (year)	58.3±13.3	60.3±12.5	0.2
Male sex [n (%)]	209(59.5)	208(59.3)	0.5
Body mass index (kg/m^2^)	23.9±3.3	24.1±3.6	0.8
Body-surface area (m^2^)	1.7±0.2	1.7±0.2	0.1
Serum creatinine (mg/dl)	1.9±1.7	1.8±1.7	0.9
Measured GFR (ml/min/1.73 m^2^)	60.7±27.9	62.8±28.1	0.7

*: Plus-minus values are means±SD.

Abbreviations: GFR, glomerular filtration rate.

#### Comparison of the performances between diabetic and non-diabetic cohorts

Bias of both the CKD-EPI equation and the Asian modified CKD-EPI equation in the non-diabetic cohort were less than those in the type 2 diabetic cohort (median difference, 2.9 and 0.3 ml/min/1.73 m^2^ vs. −3.7 and −7.3 ml/min/1.73 m^2^ [P<0.001 for both comparisons]). In terms of both precision and accuracy, GFR estimating equations all achieved better results in the non-diabetic cohort comparing with those in the type 2 diabetic cohort (IQR for difference, ranged from 20.5 to 22.2 ml/min/1.73 m^2^ for all 3 equations vs. ranged from 28.0 to 31.5 ml/min/1.73 m^2^; 30% accuracy, ranged from 64.4% to 66.7% vs. ranged from 53.0% to 57.3% [P≤0.01 for all comparisons]). However, Bias of re-expressed 4 variable MDRD equation in the non-diabetic cohort was greater than that in the type 2 diabetic cohort (median difference, 5.1 ml/min/1.73 m^2^ vs. −2.2 ml/min/1.73 m^2^ [P<0.001]) (Table 3).

**Table 3:** Performance between measured GFR and estimated GFR.

Variable	CKD group	
	Non-diabetics	Diabetics
Bias - median difference (ml/min/1.73 m^2^, 95% CI)		
Re-expressed 4 variable MDRD equation	5.1(3.2, 7.6)	−2.2(−6.8, 0.8)
CKD-EPI equation	2.9(1.3, 4.6)	−3.7(−6.7, 0.5)
Asian modified CKD-EPI equation	0.3((−1.8, 1.8)	−7.3(−10.9, −2.2)
Precision - IQR of the difference (ml/min/1.73 m^2^, 95% CI)		
Re-expressed 4 variable MDRD equation	21.2(18.4, 24.1)	31.2(25.3, 30.9)
CKD-EPI equation	20.5(17.9, 23.1)	28.0(25.3, 30.9)
Asian modified CKD-EPI equation	22.2(19.5, 25.3)	31.5(27.7, 34.1)
Accuracy - 30% accuracy (%, 95% CI)		
Re-expressed 4 variable MDRD equation	64.4(60.0, 69.2)	51.3(45.9, 56.4)
CKD-EPI equation	66.7(61.8, 71.5)	57.3(52.1, 62.1)
Asian modified CKD-EPI equation	65.0(60.1, 69.8)	53.0(47.9, 58.1)

Abbreviations: GFR, glomerular filtration rate; CKD, chronic kidney disease; MDRD, Modification of Diet in Renal Disease; CKD-EPI, Chronic Kidney Disease Epidemiology Collaboration; CI, confidence interval; IQR, interquartile range.

#### Performances of the equations in the type 2 diabetic cohort

Both the re-expressed 4 variable MDRD equation and the CKD-EPI equation appeared unbiased (median difference, −2.2 ml/min/1.73 m^2^ for the re-expressed 4 variable MDRD equation vs. −3.7 ml/min/1.73 m^2^ for the CKD-EPI equation [P = 0.5]). However, precision was improved with the CKD-EPI equation (IQR for the difference, 28.0 ml/min/1.73 m^2^), as compared with the re-expressed 4 variable MDRD equation and with the Asian modified CKD-EPI equation (IQR for the difference, 31.2 and 31.5 ml/min/1.73 m^2^), as was accuracy (30% accuracy, 57.3% vs. 51.3% and 53.0% [P = 0.004 and P = 0.01]) (Table 3).

### Development of a new modified equation to estimate GFR for type 2 diabetic patients

#### Patient's characteristics in this part of study

The general characteristics of type 2 diabetic patients are presented in Table 4. The diabetic cohort here enrolled the participants in the analyses for the first result in this paper. A total of 589 patients were enrolled, including 327 men and 260 women, and the mean age was 61.0±12.7 yr, with body mass index 24.9±4.1 kg/m^2^, fasting plasma glucose 160.4±77.0 mg/dL, glycated hemoglobin 9.3±11.9%, SC 1.4±1.5 mg/dL, and mGFR 74.4±31.0 ml/min/1.73 m^2^. We randomly selected 379 subjects (the development data set) from the entire study population, and the remaining 210 patients were included in the validation data set.

**Table 4:** Patient's characteristic.

Characteristic (N = 589)	Mean (standard deviation) or number (percentage)
Age (year)	61.0(12.7)
Male sex [n (%)]	327(55.5)
Body mass index (kg/m^2^)	24.9(4.1)
Body-surface area (m^2^)	1.7(0.2)
Serum albumin, mean (g/dL)	3.8(0.8)
Serum urea nitrogen (mg/dL)	24.0(18.0)
Serum creatinine (mg/dL)	1.4(1.5)
Serum uric acid (mg/dL)	6.4(2.3)
Serum total cholesterol (mg/dL)	199.1(85.2)
Serum triglycerides (mg/dL)	215.5(197.1)
Serum high-density lipoprotein (mg/dL)	67.2(70.5)
Serum low-density lipoprotein (mg/dL)	99.8(53.9)
Fasting plasma glucose (mg/dL)	160.4(77.0)
Glycated haemoglobin (%)	9.3(11.9)
Urine albumin to creatinine ratio (mg/mg)	57.8(132.3)
Measured GFR (ml/min/1.73 m^2^)	70.9(29.1)
GFR categories [n (%)]	
<15 (ml/min/1.73 m^2^)	13(2.2)
15–29 (ml/min/1.73 m^2^)	40(6.8)
30–59 (ml/min/1.73 m^2^)	151(25.6)
60–89 (ml/min/1.73 m^2^)	191(32.4)
>90 (ml/min/1.73 m^2^)	194(32.9)

Abbreviations: GFR, glomerular filtration rate.

#### Development of the new modified equation

We reconstructed a new modified equation using data from the development data set of this part of study by the generalized additive model. The new modified equation used the same three variables (age, gender and SC), the same knot points for SC and the same forms of smooth functions as the CKD-EPI equation. mGFR and SC were transformed to natural logarithms. The development data set was divided in to four categories according to gender and the knot points for SC. And four linear regression models were developed. The coefficients were estimated by the least-square error method (Table 1).

### Overall performance of the predicting models

In the validation data set, the new modified equation showed less bias (median difference, 2.3 ml/min/1.73 m^2^ for the new modified equation vs. ranged −3.8 to −7.9 ml/min/1.73 m^2^ for the other 3 equations [P<0.001 for all comparisons]), as was precision (IQR of the difference, 24.5 ml/min/1.73 m^2^ vs. ranged from 27.3 to 30.7 ml/min/1.73 m^2^), leading to a greater accuracy (30% accuracy, 71.4% vs. 55.2% for the re-expressed 4 variable MDRD equation and 61.0% for the Asian modified CKD-EPI equation [P = 0.001 and P = 0.02], 62.9% for the CKD-EPI equation [P = 0.4] (Table 5).

**Table 5:** Performance of bias, precision and accuracy between measured GFR and estimated GFR in the validation data set.

Variable	Measured GFR (ml/min/1.73 m^2^)		
	Overall (n = 210)	<60 (n = 86)	≥60 (n = 124)
Bias – median difference (ml/min/1.73 m^2^, 95% CI)			
Re-expressed 4 variable MDRD equation	−4.4(−8.3, −1.0)	2.3(−1.8, 5.8)	−10.7(−16.4, −6.8)
CKD-EPI equation	−3.8(−6.9, −0.2)	2.1(−1.8, 5.8)	−8.1(−11.4, −3.9)
Asian modified CKD-EPI equation	−7.9(−11.5, −3.6)	0.3(−4.1, 4.2)	−13.2(−17.1, −8.3)
New modified equation	2.3(−1.3, 5.7)	−6.5(−9.5, −4.8)	12.9(8.5, 16.4)
Precision – IQR of the difference (ml/min/1.73 m^2^, 95% CI)			
Re-expressed 4 variable MDRD equation	30.7(26.3, 36.4)	20.2(13.8, 28.6)	37.1(27.6, 49.3)
CKD-EPI equation	27.3(22,3, 30.8)	21.2(14.8, 30.2)	28.3(22.3, 33.2)
Asian modified CKD-EPI equation	29.9(25.0, 33.7)	23.4(16.8, 32.5)	29.9(23.0, 34.3)
New modified equation	24.5(21.0, 28.4)	13.6(10.2, 17.5)	23.4(18.8, 32.3)
Accuracy - 30% accuracy (%, 95% CI)			
Re-expressed 4 variable MDRD equation	55.2(48.1, 61.9^)^	41.9(31.4, 52.3)	64.5(56.5, 72.6)
CKD-EPI equation	62.9(56.2, 69.5)	44.2(33.7, 54.7)	75.8(68.6, 83.1)
Asian modified CKD-EPI equation	61.0(54.3, 67.8)	46.5(36.1, 57.0)	71.0(62.9, 79.0)
New modified equation	71.4(65.2, 77.1)	58.1(47.7, 68.6)	80.6(73.4, 87.1)

Abbreviations: GFR, glomerular filtration rate; MDRD, Modification of Diet in Renal Disease; CKD-EPI, Chronic Kidney Disease Epidemiology Collaboration; CI, confidence interval; IQR, interquartile range.

## Discussions

In the present study, we first confirmed the hypothesis that GFR estimating equations all achieved better results in the non-diabetic cohort comparing with those in the type 2 diabetic cohort by paired samples study. Then, we developed a new equation in a cohort of type 2 diabetic patients by modification of the CKD-EPI equation which performed the best in diabetic subjects. In the validation data set, the new modified equation achieved less bias, higher precision and greater accuracy compared with all three original equations. These results were consistent with the previous findings [15], [21]–[26] that modification of the original equation in a local cohort which was quiet different to the original one may improve the performance of GFR estimation in the same population. And our results will help clinicians to make suitable clinical decision for diabetic patients and avoid unnecessary examination and treatment.

Why the modified CKD-EPI equation outperformed all three original equations? There are several reasons. First, the development data set in this study was mainly type 2 diabetic patients, which was differed to the other original equations. Obesity is common in diabetic patients [27], leading to a relative low body muscle mass [28], influenced the generation of creatinine in the body. And hyperglycemia status in diabetic patients influences the measurement of GFR [29]. Second, GFR measurement method in this study was calibrated to the dual sample DTPA clearance. However, the development data sets of the other original equations used urinary clearance of iothalamate instead. Third, the validation cohort in this study had similar characters as those in the development cohort. Systematic differences generally lead to bias, whereas variation in populations' characteristics leads to imprecision.

There are limitations in our study. First, the new modified equation needs further external validations. Second, the study population in this study was restricted to Chinese patients with type 2 diabetes. Third, difference in the method to measure GFR between different equations would lead to systemic error in comparison with each other. Forth, the sample size of the validation data set in this study was relatively small. Fifth, there is not suitable statistic method for the comparison of IQR of difference between different GFR predicting models up till now.

In conclusion, we confirmed that the performances of GFR estimating equations in type 2 diabetic patients were worse than those in non-diabetic participants. And a new modified CKD-EPI equation for type 2 diabetic patients was developed and validated. The new modified equation improves the performance of GFR estimation, which may help physician to evaluate the kidney function in diabetic patients. Extensive external validations will be the next step before broadly applications.

## References

[1] Tonelli M, Muntner P, Lloyd A, Manns BJ, Klarenbach S, et al (2012) Risk of coronary events in people with chronic kidney disease compared with those with diabetes: a population-level cohort study. Lancet 380: 807–814. 22717317 10.1016/S0140-6736(12)60572-8

[2] Levey AS, Coresh J (2012) Chronic kidney disease. Lancet 379: 165–180. 21840587 10.1016/S0140-6736(11)60178-5

[3] American Diabetes Association. Standards of medical care in diabetes–2013 (2013) Diabetes Care. 36: S11–66. 10.2337/dc13-S011 PMC3537269 23264422

[4] Earley A, Miskulin D, Lamb EJ, Levey AS, Uhlig K (2012) Estimating equations for glomerular filtration rate in the era of creatinine standardization: a systematic review. Ann Intern Med 156:785–795, W–270, W–271, W–272, W–273, W–274, W–275, W–276, W–277, W–278. 10.7326/0003-4819-156-11-201203200-00391 22312131

[5] Silveiro SP, Araújo GN, Ferreira MN, Souza FD, Yamaguchi HM, et al (2011) Chronic Kidney Disease Epidemiology Collaboration (CKD-EPI) equation pronouncedly underestimates glomerular filtration rate in type 2 diabetes. Diabetes Care 34: 2353–2355. 21926286 10.2337/dc11-1282 PMC3198274

[6] Rognant N, Lemoine S, Laville M, Hadj-Aïssa A, Dubourg L (2011) Performance of the chronic kidney disease epidemiology collaboration equation to estimate glomerular filtration rate in diabetic patients. Diabetes Care 34: 1320–1322. 21540431 10.2337/dc11-0203 PMC3114318

[7] Rigalleau V, Lasseur C, Perlemoine C, Barthe N, Raffaitin C, et al (2005) Estimation of glomerular filtration rate in diabetic subjects: Cockcroft formula or modification of Diet in Renal Disease study equation? Diabetes Care 28: 838–843. 15793182 10.2337/diacare.28.4.838

[8] Nair S, Mishra V, Hayden K, Lisboa PJ, Pandya B, et al (2011) The four-variable modification of diet in renal disease formula underestimates glomerular filtration rate in obese type 2 diabetic individuals with chronic kidney disease. Diabetologia 54: 1304–1307. 21359581 10.1007/s00125-011-2085-9

[9] Rigalleau V, Lasseur C, Raffaitin C, Perlemoine C, Barthe N, et al (2006) Glucose control influences glomerular filtration rate and its prediction in diabetic subjects. Diabetes Care 29: 1491–1495. 16801567 10.2337/dc06-0407

[10] Kawamoto R, Kohara K, Tabara Y, Miki T, Ohtsuka N, et al (2008) An association between body mass index and estimated glomerular filtration rate. Hypertens Res 31: 1559–1564. 18971530 10.1291/hypres.31.1559

[11] Stevens LA, Claybon MA, Schmid CH, Chen J, Horio M, et al (2011) Evaluation of the Chronic Kidney Disease Epidemiology Collaboration equation for estimating the glomerular filtration rate in multiple ethnicities. Kidney Int 79: 555–562. 21107446 10.1038/ki.2010.462 PMC4220293

[12] Xun Liu, Huijuan Ma, Hui Huang, Cheng Wang, Hua Tang, et al (2013) Is the Chronic Kidney Disease Epidemiology Collaboration creatinine-cystatin C equation useful for glomerular filtration rate estimation in the elderly? Clinical Interventions in Aging 8: 1387–1391. 24143084 10.2147/CIA.S52774 PMC3797613

[13] National Kidney Foundation (2002) K/DOQI clinical practice guidelines for chronic kidney disease: evaluation, classification, and stratification. Am J Kidney Dis 39: S1–266. 11904577

[14] Heikkinen JO, Kuikka JT, Ahonen AK, Rautio PJ, et al (2001) Quality of dynamic radionuclide renal imaging: multicentre evaluation using a functional renal phantom. Nucl Med Commun 22: 987–995. 11505208 10.1097/00006231-200109000-00008

[15] Pei X, Yang W, Wang S, Zhu B, Wu J, et al (2013) Using mathematical algorithms to modify glomerular filtration rate estimation equations. PLoS One 8: e57852. 23472113 10.1371/journal.pone.0057852 PMC3589471

[16] Liu X, Pei X, Li N, Zhang Y, Zhang X, et al (2013) Improved glomerular filtration rate estimation by an artificial neural network. PLoS One 8: e58242. 23516450 10.1371/journal.pone.0058242 PMC3596400

[17] Xun Liu, Yanni Wang, Cheng Wang, Chenggang Shi, Cailian Cheng, et al (2013) A New Equation to Estimate Glomerular Filtration Rate in Chinese Elderly Population. PLOS ONE 8: e79675. 24244543 10.1371/journal.pone.0079675 PMC3823564

[18] Efron B, Tibshirani RJ (1993) An introduction to the bootstrap. New York: Chapman and Hall.

[19] Levey AS, Coresh J, Greene T, Stevens LA, Zhang YL, et al (2006) Chronic Kidney Disease Epidemiology Collaboration. Using standardized serum creatinine values in the modification of diet in renal disease study equation for estimating glomerular filtration rate. Ann Intern Med 145: 247–254. 16908915 10.7326/0003-4819-145-4-200608150-00004

[20] Levey AS, Stevens LA, Schmid CH, Zhang YL, Castro AF 3rd, et al (2009) A New Equation to Estimate Glomerular Filtration Rate. Ann Intern Med 150: 604–612. 19414839 10.7326/0003-4819-150-9-200905050-00006 PMC2763564

[21] Teo BW, Xu H, Wang D, Li J, Sinha AK, et al (2011) GFR estimating equations in a multiethnic Asian population. Am J Kidney Dis 58: 56–63. 21601325 10.1053/j.ajkd.2011.02.393

[22] Lee CS, Cha RH, Lim YH, Kim H, Song KH, et al (2010) Ethnic coefficients for glomerular filtration rate estimation by the Modification of Diet in Renal Disease study equations in the Korean population. J Korean Med Sci25: 1616–1625. 10.3346/jkms.2010.25.11.1616 PMC2966999 21060751

[23] Matsuo S, Imai E, Horio M, Yasuda Y, Tomita K, et al (2009) Revised Equations for Estimating Glomerular Filtration Rate (GFR) form Serum Creatinine in Japan. Am J Kidney Dis 53: 982–992. 19339088 10.1053/j.ajkd.2008.12.034

[24] Praditpornsilpa K, Townamchai N, Chaiwatanarat T, Tiranathanagul K, Katawatin P, et al (2011) The need for robust validation for MDRD-based glomerular filtration rate estimation in various CKD populations. Nephrol Dial Transplant 26: 2780–2785. 21357214 10.1093/ndt/gfq815

[25] Horio M, Imai E, Yasuda Y, Watanabe T, Matsuo S, et al (2010) Modification of the CKD epidemiology collaboration(CKD-EPI) equation for Japanese: accuracy and use for population estimates. Am J Kidney Dis 56: 32–38. 20416999 10.1053/j.ajkd.2010.02.344

[26] van Deventer HE, George JA, Paiker JE, Becker PJ, Katz IJ (2008) Estimating glomerular filtration rate in black South Africans by use of the modification of diet in renal disease and Cockcroft-Gault equations. Clin Chem 54: 1197–1202. 18487286 10.1373/clinchem.2007.099085

[27] de Boer IH, Sibley SD, Kestenbaum B, Sampson JN, Young B, Cleary PA, et al (2007) Central obesity, incident microalbuminuria, and change in creatinine clearance in the epidemiology of diabetes interventions and complications study. J Am Soc Nephrol 18: 235–43. 17151331 10.1681/ASN.2006040394 PMC2622719

[28] Kim TN, Park MS, Kim YJ, Lee EJ, Kim MK, et al (2014) Association of low muscle mass and combined low muscle mass and visceral obesity with low cardiorespiratory fitness. PLoS One 9: e100118. 24937121 10.1371/journal.pone.0100118 PMC4061126

[29] Bjornstad P, McQueen RB, Snell-Bergeon JK, Cherney D, Pyle L, et al (2014) Fasting blood glucose—a missing variable for GFR-estimation in type 1 diabetes? PLoS One 9: e96264. 24781861 10.1371/journal.pone.0096264 PMC4004575

